# Role of Protein Kinase CK2 in Aberrant Lipid Metabolism in Cancer

**DOI:** 10.3390/ph13100292

**Published:** 2020-10-05

**Authors:** Barbara Guerra, Olaf-Georg Issinger

**Affiliations:** Department of Biochemistry and Molecular Biology, University of Southern Denmark, 5230 Odense, Denmark; ogi@bmb.sdu.dk

**Keywords:** lipogenesis, adipogenesis, metabolic changes in cancer, protein kinase CK2

## Abstract

Uncontrolled proliferation is a feature defining cancer and it is linked to the ability of cancer cells to effectively adapt their metabolic needs in response to a harsh tumor environment. Metabolic reprogramming is considered a hallmark of cancer and includes increased glucose uptake and processing, and increased glutamine utilization, but also the deregulation of lipid and cholesterol-associated signal transduction, as highlighted in recent years. In the first part of the review, we will (i) provide an overview of the major types of lipids found in eukaryotic cells and their importance as mediators of intracellular signaling pathways (ii) analyze the main metabolic changes occurring in cancer development and the role of oncogenic signaling in supporting aberrant lipid metabolism and (iii) discuss combination strategies as powerful new approaches to cancer treatment. The second part of the review will address the emerging role of CK2, a conserved serine/threonine protein kinase, in lipid homeostasis with an emphasis regarding its function in lipogenesis and adipogenesis. Evidence will be provided that CK2 regulates these processes at multiple levels. This suggests that its pharmacological inhibition combined with dietary restrictions and/or inhibitors of metabolic targets could represent an effective way to undermine the dependency of cancer cells on lipids to interfere with tumor progression.

## 1. Introduction

Lipids are not only major components of biological membranes and a source of energy for the organism, but also messenger molecules taking part in many intracellular signaling pathways. Lipid metabolism is a vast topic covered in detail by excellent reviews and books for which a selection is cited here [1,2,3]. The purpose of this introduction is to provide an overview of the main types of lipids and discuss their major biological functions in eukaryotic cells. This is in order to fully appreciate their role in cancer development and how cellular signaling mediated by selected pro-survival protein kinases contributes to the regulation of aberrant lipid metabolism in malignant cells.

## 2. Major Types of Lipids: Their Biological Function and Regulation—An Overview

Lipids are biomolecules whose function is to store energy, be part of intracellular signaling cascades and an important component of cell membranes. The three major types of lipids found in membranes are glycerophospholipids, sphingolipids and cholesterol. Glycerophospholipids and sphingomyelins, which are a type of sphingolipids, are also known simply as phospholipids. Phospholipid molecules play an important role as second messengers [4], their function in cell signaling events will be further discussed in this review later on.

The lipid composition of biological membranes varies according to the specificity of the tissues and even within a single cell; the percentage of lipid components of membranes that are part of different organelles can vary significantly. The plasma membrane contains 40–90% of total cholesterol while the endoplasmic reticulum contains just a small fraction of this lipid (e.g., approximately 0.5% of total cholesterol in human fibroblasts) [5]. Intracellular membranes, such as the nuclear and the outer/inner mitochondrial membranes, primarily contain glycerophospholipids.

The ability of lipids to store energy is mainly represented by what is commonly known as fat and includes molecules such as fatty acids and their derivatives. The storage form of fatty acids is represented by triacylglycerols (TAGs) where all three hydroxyl groups on glycerol are esterified with a fatty acid. Most fatty acids are provided through the diet; however, complex biochemical reactions ensure their de novo synthesis from small molecules [2]. Acetyl-CoA is the priming molecule from which fatty acids begin to be formed. Acetyl-CoA undergoes carboxylation to form malonyl-CoA. This reaction is catalyzed by acetyl-CoA carboxylase (ACC) a key enzyme in the synthesis of the first saturated fatty acid (i.e., palmitic acid) and that is regulated by the serine/threonine kinase AMP-activated protein kinase (AMPK) [6]. All other known fatty acids are synthesized from palmitic acid in the presence of fatty acid synthase (FASN) where acetyl-CoA represents the carbon atoms source for their synthesis. The degree of utilization of fatty acids varies significantly from tissue to tissue and depends on the metabolic status of the body.

Energy producing pathways in eukaryotes require the intervention of a number of enzymes and proteins that are tightly regulated by post-translational modifications but also subjected to epigenetic control. The importance of epigenetic regulation is supported by studies with pancreatic cancer cells where up-regulation of ATP citrate lyase (ACLY), an enzyme that contributes to the synthesis of acetyl-CoA, results in high levels of acetyl-CoA. This, in turn, favors malignant progression through the mevalonate pathway and the AKT-ACLY signaling cascade [7]. Enhanced levels of acetyl-CoA have also been shown to induce metabolic reprogramming of cells through alterations of glycolytic enzymes as a result of increased acetylation of histone proteins and, thus, up-regulation of gene expression [8].

Overall, these findings support the notion that acetyl-CoA should not be considered as just a passive donor of acetyl groups but rather an important signaling molecule controlling cell metabolism.

### Lipids in Signal Transduction

Phospholipids in membranes play a variety of roles as structural components and as second messengers in signal transduction [4]. Phospholipids are polar lipids composed of glycerol, two fatty acids, a phosphate group and a polar group such as choline, serine or ethanolamine. Phosphatidylcholine, phosphatidylethanolamine and phosphatidylserine are major components of all cellular membranes and can form bilayers. One of the most important factors is phosphatidylinositol 4,5-bisphosphate (PIP_2_) that is an inositol-containing phospholipid. PIP_2_ is the precursor of 1,2-diacyglycerol (DAG) and inositol 1,4,5-trisphosphate (IP_3_). These molecules are formed in the presence of phospholipase C (PLC) and serve as second messengers in cell signaling triggering the release of calcium from deposits in the endoplasmic reticulum (i.e., IP_3_) and stimulating the activity of protein kinase C (i.e., DAG), respectively [9,10]. Independent work from two research groups led to the identification of a novel protein kinase (i.e., PI3K) able to catalyze the phosphorylation of inositol phospholipids on the 3′ position of the inositol molecule (PtdIns(3)P) in fibroblasts stimulated with platelet-derived growth factor (PDGF) [11,12,13] and a novel phosphoinositide containing four phosphate groups (PtdInsP_3_ or PIP_3_) found in activated neutrophils from human donors [14]. The formation of PIP_3_ is pivotal for the activation of signaling cascades, which confer pro-survival, and growth signals to the cell. Proteins such as phosphoinositide-dependent kinase 1 (PDK1), AKT/PKB, receptor of phosphoinositides 1 (GRP-1), cytohesin- 2 (ARNO), centaurin-1, cytohesin-1, and Bruton’s tyrosine kinase (BTK) are able to migrate to the plasma membrane and bind with high selectivity PIP_3_ via their Pleckstrin Homology (PH) domain [15,16]. Activation of a specific protein kinase may not exclusively require its assembly to the cell membrane via binding of the PH domain to PIP_3_. In some cases, PIP_3_ interaction with PH domains may happen in the absence of recruitment to the plasma membrane and this latter event occurs by protein-protein interaction and through a PIP_3_-independent mechanism [17]. PI3K and AKT/PKB are likely the most important protein kinases activated by the presence of PIP_3_. Indeed, the pathway they control plays a major role in cancer with respect to cell survival, growth and metastasis [18].

De-activation of the PI3K-AKT signaling cascade is controlled by the PIP_3_ 3-phosphatase, Phosphatase and Tensin Homology (PTEN), which rapidly removes the 3-phosphate resulting in the conversion of PIP_3_ into PIP_2._ However, removal of 5-phosphate is also possible by the SH2-containing inositol 5′-phosphatase (SHIP) and synaptojanin phosphatases although the resulting effect is not quite the same. In fact, while the activity of PTEN reduces the concentration of PIP_3_ and attenuates the PI3K-AKT signaling pathway, the consequence of the 5-phosphatases action seems less clear since the resulting product is still a lipid able to bind proteins through their PH domain [16,19,20,21]. The PH domain is not the only element that allows the binding of a protein kinase to membrane lipids [22]. Some kinases such as serum glucocorticoids kinase-3 (SGK-3) are equipped with a related PX domain [23] while other lipid-kinases employ the C2 domain. This is the case of conventional protein kinase C (PKC), which binds anionic phospholipids in the presence of calcium [24,25]. Interestingly, PDK1 phosphorylates both AKT and PKC, however, while PDK1-catalyzed phosphorylation of PKC represents a maturation step for PKC, phosphorylation of AKT directly activates this kinase [26].

Overall, lipid-controlled protein kinases together with specific lipid-second messengers play a major role in key intracellular signal transduction pathways despite the fact that they represent only approximately 10% of the entire kinome and, yet, their de-regulation may have detrimental effects for the cell.

The vast field of lipid signal transduction would not be complete without mentioning the crosstalk between phospholipids and phospholipases. Phospholipases are grouped in several families; i.e., PLA, PLC and PLD. Phospholipase A (PLA) isoforms, which include both secretory and cytosolic forms, represent the largest family [27]. These enzymes cleave membrane phospholipids resulting in one free fatty acid. The most physiologically important substrate for PLA_2_ is phosphatidylcholine, its cleavage leads to production of eicosatetraenoic acid (arachidonic acid, AA), which is the substrate for three intracellular pathways: i.e., the cyclooxygenase (COX)-, lipooxigenase (LOX)- and cytochrome P-450 2C (CYP2C)-mediated cascades. The reactions catalyzed by COXs give rise to a series of compounds including prostaglandins (PGs) and thromboxanes (TXs) that are natural mediators of inflammation [28], reproduction [29] and gastric acid secretion [30]. The action of LOX isoforms results in the synthesis of leukotrienes and other lipid messengers involved in the control of respiration [31] while CYP2C catalyzes reaction leading to epoxyeicosatrienoic acids (EETs) [32,33].

The PLA isoforms represent the most abundant family of phospholipases, nonetheless, PLC is the most known type of phospholipase. As mentioned earlier, this enzyme catalyzes the hydrolysis of PIP_2_ to form DAG and IP_3_ that are second messengers involved in calcium-mediated signaling (i.e., IP_3_) and activation of PKC (i.e., DAG) [9,10]. PKC has long been known for its role in signal transduction and given the plethora of substrates, a multiplicity of functions has been attributed to this enzyme including receptor desensitization, transcriptional regulation, cell growth, memory and learning modulation. Perhaps one of the most interesting properties of PKC in the context of cancer is its involvement in adaptive immune response in mammals regulating lymphocytes adhesion, migration, differentiation and proliferation making this protein kinase a defense molecule in eukaryotic organisms. These and other functions of PKC are described in several excellent reviews [34,35,36,37].

## 3. Metabolic Changes in Cancer Development

### 3.1. Lipid Metabolism in Cancer Cells

In mammals, the production of lipids, which includes de novo fatty acids (FAs) and cholesterol synthesis, takes place mainly in the liver and adipose tissue through complex and energy-consuming biochemical reactions. The synthesis of lipids in healthy organisms is tightly controlled and responds to the nutrient’s status of the cell. However, many human cancers display aberrant lipid metabolism which includes not only de novo lipogenesis but also alterations in fatty acids transport and storage as liquid droplets [38]. Because of the important role of FAs in many aspects of carcinogenesis, the identification of chemical inhibitors targeting FA metabolism has become an emerging research focus [39].

**Figure 1:**
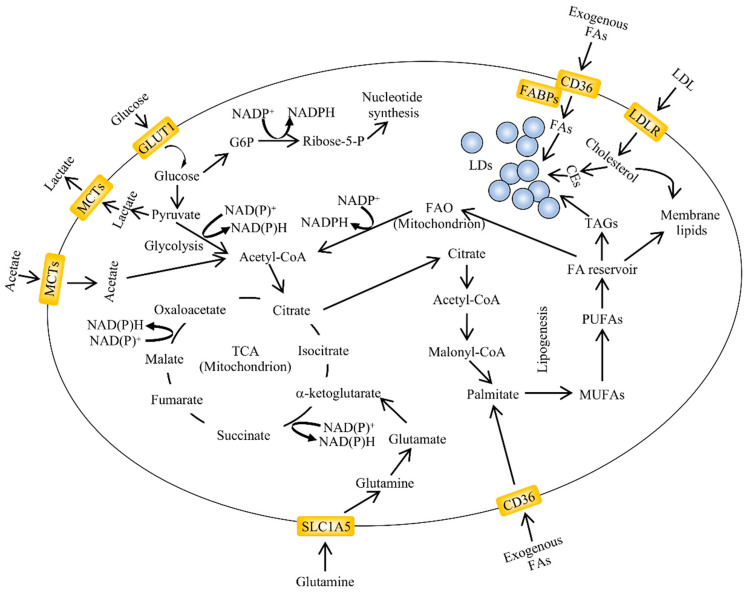
An overview of aberrant lipid metabolism in cancer. Alterations in lipid metabolism are found in many types of cancers. The major pathways regulating lipid accumulation are de novo lipogenesis, lipid uptake and fatty acid oxidation (FAO). The exogenous uptake of fatty acids can be facilitated by membrane transporters such as cluster of differentiation 36 (CD36). Lipid droplets (LDs) are organelles which contain variable ratios of neutral lipids such as cholesteryl esters and triglycerides with saturated or unsaturated chains. Their content can be used for energy and acetyl-CoA production through fatty acid oxidation in the mitochondria. The carbon source for de novo lipogenesis in cancer cells is represented by glucose, glutamine and acetate (adapted from [46]). CEs, cholesteryl esters; FABPs, fatty acid-binding protein; GLUT1, glucose transporter 1; G6P, glucose-6-phosphate; LDL, low density lipoprotein; LDLR, low density lipoprotein receptor; NADPH, nicotinamide adenine dinucleotide phosphate—reduced form; NADP^+^, nicotinamide adenine dinucleotide phosphate—oxidized form; MCTs, monocarboxylate transporter; MUFAs, monounsaturated fatty acids; PUFAs, polyunsaturated fatty acids; SLC1A5, solute carrier family 1 member 5; TCA, tricarboxylic acid cycle.

Increased lipogenesis is supported by enhanced expression and/or activity of lipogenic enzymes such as pyruvate dehydrogenase (PDH), ACC, FASN, stearoyl-CoA desaturase (SCD) and 3-hydroxy-3-methyl-glutaryl-CoA reductase (HMG-CoA) [40,41]. Metabolic reprogramming in cancer cells was first reported in 1924 when Otto Warburg and co-workers demonstrated alterations in glucose metabolism resulting in aerobic glycolysis to support the high growth rates of transformed cells [42]. While aberrant glucose metabolism was mistakenly attributed to defects in the function of mitochondria, it is generally accepted that increased glycolysis, even in the presence of oxygen, provides intermediates for the biosynthesis of nucleosides and amino acids. These, in turn, support the production of macromolecules and organelles required for active cell proliferation (Figure 1, [43,44,45]).

Genomic and transcriptomic data derived from over thousands tumor samples across different types of cancer revealed that mutations on specific driver genes are the main force responsible for the development of cancer cells and drive a selection process which primarily converges on a metabolic network of reactions [47]. Oncogenic hotspot mutations may also affect metabolic enzymes. For example, FASN up-regulation and increased activity represent one of the most frequent phenotypic alterations in cancer cells. Extremely high levels of this enzyme are found in many human epithelial cancers including breast, colorectal, prostate, bladder, melanoma and stomach [48]. Marked increases in ACLY expression and activity have been found in many types of cancer cells and inhibition of this enzyme has been shown to reduce tumorigenesis in vivo [49,50]. Overexpression of ACC is often reported in advanced breast cancer and pre-neoplastic lesions and associated with increased risk of infiltrating breast cancer and reduced survival of patients [51,52]. Traditionally, cancer research has primarily focused on the glycolytic pathway, glutaminolysis and fatty acid synthesis. However, studies in the past ten years have started to also highlight the important contribution of fatty acid oxidation for the function of cancer cells suggesting a context-dependent regulation of lipid metabolism [45]. Enzymes controlling fatty acid oxidation are highly expressed in colorectal, hepatic and prostate cancers, and it has been suggested that this process might be an important bioenergetic source for ATP and NADPH [45,53]. The study of fatty oxidation in cancer cells has given an exciting new dimension to cancer research mining the dogma that the synthesis of fatty acids is not compatible with their oxidation and providing, beyond any doubt, a novel therapeutic opportunity in cancer.

### 3.2. Oncogenic Signaling Supports Aberrant Lipid Metabolism

The observation that lipogenic enzymes are overexpressed both at the mRNA and protein levels in cancer led to the notion that excessive growth factor signaling contributes to the de-regulated expression of lipogenic enzymes. Overexpression of the human epidermal growth factor receptor 2 (HER2) has been shown to contribute to up-regulation of de novo lipogenesis and the aggressiveness of cancer cells by stimulating the PI3K signaling [54]. Activation of AKT also contributes to lipid metabolism in cancer cells. For instance, this enzyme (i) phosphorylates and activates ACLY [50,55], (ii) can directly activate the nuclear factor-like 2 transcription factor resulting in the transactivation of genes involved in the synthesis of NADPH an essential co-factor in anabolic processes [56], and (iii) suppresses β-oxidation by decreasing the expression levels of carnitine acyltransferase 1A the enzyme catalyzing the formation of acyl-carnitine [57].

The PI3K-AKT signaling cascade is closely connected to the mammalian target of rapamycin (mTOR) pathway. mTOR is a serine/threonine protein kinase which can form two multi-protein complexes (i.e., mTORC1 and mTORC2) that are distinguished by the type of interacting proteins [58]. The mTOR pathway is one of the major nutrient-sensitive cascades regulating growth, metabolism, the aging process and diseases like cancer and epilepsy, in mammals [59]. mTORC1 is considered to be a key integrator of growth factors and nutrient-mediated signals whose activation culminates in anabolic processes such as protein synthesis and metabolism [60,61]. However, unlike mTORC2, mTORC1 alone is insufficient to stimulate lipid synthesis without functional AKT [62]. In contrast to this, mTORC2 plays a pivotal role in FA metabolism through activation of downstream kinases including AKT, SGK and PKC [18,63,64,65]. In support of this, inhibition of mTORC2, but not mTORC1, has been shown to reduce the expression of genes regulating de novo lipogenesis and lipid content in hepatocellular carcinoma in vivo [66].

The regulation of de novo lipogenesis occurs mainly at the transcriptional level and involves the activation of sterol regulatory element-binding proteins (SREBPs) that are basic helix-loop-helix transcription factors regulating the synthesis of cholesterol and FAs [67]. SREBP1a and SREBP1c result from the alternative splicing of the *SREBPF1* gene while SREBP2 is encoded by the *SREBPF2* gene [68]. In mammals, SREBP1 is mainly involved in the regulation of genes coding for proteins involved in FAs synthesis while SREBP2 controls the synthesis of cholesterol. The PI3K-AKT-mTORC1 signaling cascade regulates the maturation of SREBP1. The inhibitory phosphorylation of the phosphatidic acid phosphatase lipin 1 (LPIN1), which is a negative regulator of nuclear SREBP1, by mTORC1 leads to induction of SREBP1 thereby promoting expression of its target genes including FASN, ACLY, ACC and SCD1 [69,70]. Since lipogenesis is one of the key processes supporting the proliferative status of cancer cells, it is not surprising that the activity of SREBP1 is higher in cancer cells than in normal tissues and that pharmacological targeting of this transcription factor can have a favorable response in the treatment of cancer. This is supported by in vivo studies employing EGFRvIII-bearing glioblastoma cells showing the high dependency of the cells on SREBP1 for survival and demonstrating that inhibition of SREBP1 and FASN, respectively, promotes massive tumor cell death in cancer cells bearing activated EGFR signaling [71].

AMPK is an intracellular energy sensor. This function has been consolidated by many studies demonstrating AMPK’s role in metabolic diseases such as type-2 Diabetes and obesity but also in cancer because of its unique ability to regulate cancer cell proliferation through reprogramming of cell metabolism [72,73,74,75]. AMPK was originally shown to be activated in response to ATP deprivation, more recently, it has been suggested that the activity of this enzyme may be regulated by tissue specific ubiquitination, phosphorylation and calcium levels in the context of energy homeostasis [76]. AMPK inhibits lipogenesis and cholesterol synthesis by targeting the expression and/or activity of key lipogenic enzymes such as ACC and HMG-CoA but also by negatively modulating the activity of SREBP1c and carbohydrate response element-binding protein (ChREBP) transcription factors. This results in the marked inhibition of the genes coding for ACC and FASN expression in the liver [73,77,78,79]. One of the first pieces of evidence linking AMPK to cancer was derived from studies showing that liver kinase B1 (LKB1) inhibits mTORC1 through AMPK [74]. However, in contrast to previous expectations, the role of AMPK as a tumor suppressor turned out to be not so straightforward. Substantial evidence points to a key function of AMPK in the metabolic reprogramming of cancer cells. Emerging evidence suggests, in fact, that several oncogenic signaling molecules such as oncogenic Src, Myc and H-Ras can activate AMPK, although the mechanisms involved are not always well defined [80,81,82].

Overall, although AMPK was initially suggested as a tumor suppressor, there is ample evidence that this enzyme could be, instead, a tumor promoter [83,84]. This notion is based on its ability to control the metabolic reprogramming of cancer cells under stress circumstances as those occurring in the tumor microenvironment. If so, the identification of selective and potent inhibitors of AMPK could turn out to be promising compounds in anti-cancer therapies.

### 3.3. Symmetric Metabolic Reprogramming among Cancer Types with One Exception

Metabolic reprogramming is a crucial process in cancer development and considered one of the hallmarks of malignant transformation [43]. It includes not only alterations in lipid metabolism but also elevation in glycolysis, glutaminolytic flux, amino acid metabolism, mitochondria biogenesis and pentose phosphate pathway. Although it is a phenomenon resulting from oncogenic mutations, the metabolic switch observed in the majority of cancer types seems to be context-dependent, namely, largely attributable to the microenvironment, rather than specific to the particular type of malignancy [85,86]. An interest notion that emerged in recent years is that most cancer samples retain a substantial similarity with the tissue of origin regarding the expression of metabolic genes. This may have important therapeutic implications, since targeting tumor metabolism could lead to elevated toxicity given the extensive overlapping of metabolic networks in normal as well as in tumor tissues. Apart from the fact that metabolic regulation is substantially similar between the tumor and the normal tissue from which the malignancy derived, the analysis of gene expression changes across 13 different types of primary tumors has revealed that oncogenic mutations independently contribute to the deregulation of cell metabolism; however, they all seem to influence a common sub-set of pathways converging to the control of energy nucleotides and lipid metabolism [87]. Despite this striking similarity across different types of cancers, this symmetry is largely compromised in kidney cancer, particularly, in clear cell renal cell carcinoma (ccRCC) [88]. Here, the analysis of metabolic gene expression profiles revealed that the loss of the von Hippel-Lindau (*VHL*) tumor suppressor gene and key metabolic genes located next to *VHL* results in a ccRCC-specific set of genetic aberrations shaping a unique metabolic reprogramming which supports cancer progression [88]. Such reprogramming results in the repression of nucleotide, alanine, aspartate, and glutamate metabolism, that is generally enhanced in most cancer types. Interestingly, ccRCC seems to adapt to these defective networks by upregulating signal transducer and activator of transcription 1 (STAT1), which may contribute to stimulate complementary genes involved in nucleotide biosynthesis and inositol metabolism [88].

ccRCC is the most common form of kidney cancer composed of malignant epithelial cells with clear cytoplasm due to the huge accumulation of glycogen and lipids. A comprehensive molecular characterization of ccRCC revealed unique signatures that could explain the high vasculature and histological features observed in this type of cancer. The uniqueness of ccRCC metabolic networks has to be ascribed to the loss of chromosome 3p encompassing all of the four commonly mutated genes (i.e., *VHL*, *PBRMI*, *BAP1* and *SETD2*) leading to alterations in genes coding for chromatin remodeling proteins and in genes controlling cellular oxygen sensing [89]. Genetic loss on chromosome 14 is also frequently observed and this is associated with the loss of *HIF-1A*, which unlike *HIF-2A* has been predicted to be a ccRCC tumor suppressor gene product at least in certain conditions [90]. Interestingly, the analysis of genetic programs altered in ccRCC when cells are exposed to protocols inducing adipogenic differentiation revealed a propensity of ccRCC to lipid deposition rather than lipid catabolism preserving the ability to proliferate a feature certainly uncommon in differentiating human adipocytes [91]. Excessive lipid accumulation is one of the hallmarks in ccRCC, however, this phenotype is observed in other malignancies including Burkitts lymphoma, hepatocellular carcinoma and advanced prostate cancer [92,93]. What is strikingly observed in ccRCC is that 14 metabolic genes displayed a simultaneous loss of heterozygosity (compared to the normal kidney) in regions located on chromosome 3p affecting the expression of genes regulating glycerophospholipids metabolism, oxidative phosphorylation, nucleotide and inositol phosphate metabolism. Recent work by Du et al., showed that the gene coding for carnitine palmitoyltransferase 1A (i.e., *CPT1A*) which is the rate-limiting enzyme of the FAs transport system controlling their entry into the mitochondria, is a hypoxia (i.e., HIF)-target gene. *CPT1A* expression appears repressed by HIF and this results in reduced transport of fatty acids into the mitochondria and increased lipid deposition, thus, increasing the catalogue of genes that support the propensity of ccRCC to accumulate lipids for storage rather than accelerate their cellular metabolism [94]. It remains to be determined whether excessive accumulation of lipids is a consequence of the genetic alterations occurring in ccRCC or whether changes in lipid metabolism which result in lipid droplets accumulation, promotes an escalating malignancy of the cells. Although not fully understood at present, work by Du and colleagues points towards a role of CPT1A in contributing to ccRCC development as its re-establishment into VHL-defective cells blocks lipid accumulation and, most importantly, tumorigenesis [94]. Recent work by Bensaad et al. also provided compelling evidence that lipid accumulation confers a selective growth advantage to cancer cells. They showed that increased lipid accumulation in breast cancer and glioblastoma cells is due to up-regulation of fatty acid uptake via HIF-1-mediated transactivation of FABP3 and FABP7 fatty acid binding proteins while inhibition of lipid storage reduces protection from hypoxia, the survival of cells and strongly impairs tumorigenesis in vivo [95]. Overall, while it remains to be seen whether the role of the aforementioned genes is limited to the types of cancers employed in these studies, the growing knowledge on lipid metabolism deregulation and signaling molecules controlling the involved processes might undoubtedly be able to offer novel and promising pharmacological approaches for cancer therapy.

### 3.4. The Regulatory Role of HIFs in Lipid Metabolism

Lack of oxygen in cells and tissues leads to reprogramming of gene expression coordinated by the hypoxia inducible factors (HIFs) a family of enzymes which consists of three α-subunits (HIF-1α, HIF-2α and HIF-3α) and one β-subunit (HIF-1β) [96]. The role of HIF-1 in cancer and inflammation is well established [97]. Beside the involvement of HIF-1 activation in carbohydrate metabolism, more recently, it has been shown that this transcription factor has effects on lipid metabolism. This role is particularly important in cancer in order to maintain the high proliferation rate observed in malignant cells. Gene reprogramming in lipid metabolism under hypoxia results in enhanced lipogenesis by modulation of proteins controlling fatty acids synthesis, storage and uptake. The subsequent accumulation of lipids leads to inhibition of enzymes controlling fatty acid degradation [98]. The essential role of HIFs in regulating lipid metabolism is supported by studies involving many cancer types showing that the down-regulation of HIFs or HIFs-dependent genes controlling lipid accumulation, results in a reduction in cell proliferation and chemoresistance [99,100,101]. HIF-dependent regulation of lipid metabolism has also been reported in obesity and obesity-related diseases. Adipose tissue from obese people is characterized by a high expression of HIF-1α [102], and the activation of HIF-1α induces obesity [103]. In contrast to the above, there have been in vivo studies showing that obesity is increased by the inhibition of HIFs [104]. Hence, it appears that HIFs can function as suppressors or promoters of obesity, depending on different metabolic states. Data so far have shown that the involvement of HIFs in obesity is conflicting. It is very likely that the genetic background, age and diet of the experimental animals used in these studies may account for the different results reported [98].

### 3.5. Nutrients Deprivation as a Strategy for Cancer Therapy

Cancer cells arise from genetic or epigenetic abnormalities affecting both coding and regulatory regions of the genome. They have a much higher nutrient demand than normal tissues [105], therefore, different inhibitors have been tested for anti-cancer activities. Beside calorie restriction, fast-mimicking diets and the ketogenic diet, amino acids glutamine, asparagine and arginine were chosen for starvation in cancer treatment [106]. In normal tissues, most of the pyruvate formed derives from glycolysis via the TCA cycle and is oxidized via oxidative phosphorylation. By contrast in tumor cells the pyruvate is largely converted to lactic acid and energy is produced anaerobically [107]. Differentiated tissue produces in the presence of oxygen through oxidative phosphorylation ca. 36 mol ATP/mol glucose. Under aerobic glycolysis only 2 mol ATP/ mol glucose are formed. By contrast, in proliferating tissue and in tumor cells, 4 mol ATP/mol glucose are formed through aerobic glycolysis.

One could believe that tumor cells do not need much ATP, or that normal oxidative phosphorylation is disturbed. Hence, more glycolysis is needed. Indeed, tumor cells need large amounts of sugar. Hence, the reduction in sugar will put the tumor cells into problems. Lack of sugar is the Achilles heel for tumor cells. Glucose and amino acids, especially glutamine, are highly demanded nutrients in cancer cells. Besides the already mentioned diet restriction strategies to starve cancer cells, there is also the possibility to interfere with glutamine metabolism. The basis for this is the conversion of glutamine into alpha-ketoglutarate for ATP production in oxidative phosphorylation to provide energy for the cells. One option to interfere with cancer cells growth is to stop from obtaining glutamine. This can be achieved by glutamine depletion, glutaminase inhibition and membrane glutamine transporter inhibition [108,109,110,111,112]. The most ideal target would be an amino acid for which cancer cells but not normal cells are highly dependent [106]. Several cancers have been found that are auxotrophic for non-essential amino acids. In a number of cases, this has been achieved through loss of expression of an enzyme involved in their synthesis [113,114,115].

The energy requirement, i.e., ATP production, is one side of the coin; the need for sugar is the other side. It will be interesting to see how different tumors with respect to their sugar demand, behave when the ATP production is down-regulated, e.g., via glutamine starvation.

A correlation between food intake and cancer has been observed already over a hundred years ago [116]. The author observed during tumor transplantation studies in mice that the growth of grafts of transplantable tumors could be, in many cases, prevented or retarded by calorie restriction. The preliminary observations have been substantiated over the years in animal experiments [117]. In his studies, Tannenbaum investigated the salutary action of underfeeding or calorie restriction on longevity. Tumor growth was retarded and led to longer survival of the host. Moreover, tumors metastasized with only moderate frequency. In mice, fasting affects the growth of a number of cancers, similarly to chemotherapy. Hence, the application of both treatments can result in cancer-free survival [118,119]. Fasting can lead to the prevention of chemotherapy-induced DNA damage in healthy tissues and helps to maintain patient quality of life during chemotherapy [120,121,122]. Moreover, fasting, calorie restriction and ketogenic diet affect the response to anti-cancer therapy, especially leading to reduced Akt/mTOR and Ras signaling in normal cells. Growth is reduced. By contrast, in tumor cells, Akt/mTOR and Ras/mitogen-activated protein kinase (MAPK) signaling is enhanced and so is growth. In systemic circulation, fasting leads to reduced substrate availability, reduced growth factors and reduced inflammation, whereas in the tumor environment enhanced drug delivery and increased tumor clearance is observed [123]. Calorie restriction extends lifespan. Recent studies have tested the potential of calorie restriction as an adjuvant therapy to enhance the efficacy of chemotherapy, radiation therapy, and novel immunotherapies [124,125,126,127]. Ongoing and future clinical studies will indicate whether conventional treatments and calorie restriction will prove to be a promising approach in cancer therapy.

### 3.6. Is Cancer a Metabolic Disease?

The metabolic characteristics of tumors are essential for survival; their targeting could have an essential effect on tumor viability. However, as we have seen in the case of interfering with proliferation, there are severe side effects to be expected since a drug directed against a certain metabolic pathway in a tumor would also affect the same pathway in non-tumorigenic cells. It is foreseeable that immune cells could be particularly susceptible to anti-cancer therapies, which is quite concerning as they are the cells that would normally target the tumor. Nevertheless, there is some reason to be hopeful about the prospects of metabolic targeting as a combination of inhibitors targeting energy metabolism with other antitumor drugs could represent a powerful new therapeutic approach [128,129]. Cancer cells are characterized by a reprogramming of energy metabolism. Therefore, understanding the metabolic changes that occur in cancer is absolutely mandatory for the successful interference with cancer metabolism.

Taking everything into consideration, a combination of various approaches, as described above, will certainly have the potential to tackle cancer more specifically than chemotherapy alone, with the prospect of less side effects, thereby ameliorating the cancer patients’ life quality.

## 4. The Role of CK2 in Lipid Metabolism

### 4.1. Background

CK2 is a constitutively active and evolutionary conserved serine/threonine protein kinase that has been linked to the regulation of many intracellular processes and diseases, particularly cancer, through the phosphorylation and/or its association with specific substrate targets. In mammals, CK2 can be expressed as a tetrameric holoenzyme composed of two catalytic isoforms (CK2α and/or CK2α’) and two regulatory isoforms (CK2β) [130,131]. However, compelling evidence has indicated that the individual isoforms can exert functions on their own and display different subcellular localizations challenging the traditional view of CK2 as a stable tetrameric enzyme. Many features defining this enzyme at the molecular level cannot be covered in this review. However, excellent reviews exploring different aspects of this protein kinase have been published; some are cited here [132,133,134,135,136,137,138].

The link between the upregulation of CK2 and cancer is well established. One of the first reports on increased protein kinase CK2 activity and elevated expression in tumor tissue as compared to the non-neoplastic counterpart from the same patient, was from Muenstermann et al. [139]. Seitz et al. [140] had already shown enhanced CK2 activity in colonic carcinomas after heterotransplantation in nude mice a year earlier. Several studies followed, showing elevated CK2 activity and expression levels in colorectal mucosa, adenomas, carcinomas and in human kidney tumors (reviewed in [134]). These very first reports were supported by a plethora of publications in the following years [141,142,143,144,145,146], including several excellent reviews summarizing the important contribution of CK2 in cancer [132,133,134,136,147,148,149,150]. Collectively, these studies identified CK2 as a scientifically validated cancer target that remained therapeutically unexploited until the first highly specific inhibitor CX-4945 (i.e., Simitasertib) was identified [151]. In recent years, CX-4945 has been employed in Phase I, I/II clinical trials in patients with advanced solid cancers, Castelman’2 disease or multiple myeloma (test IDs: NCT00891280, NCT01199718), cholangiocarcinoma (test ID: NCT02128282), carcinoma, basal cell (test ID: NCT03897036) or pediatric medulloblastoma (test ID: NCT03904862). Although the outcome of these tests is still pending, pre-clinical studies with CX-4945 have shown encouraging results providing evidence of clinical potential in trials involving humans.

In light of the emerging role of metabolic enzymes and reprogramming of lipid metabolism in cancer development, we will examine in this review the potential crosstalk between CK2 and lipid metabolism and how this enzyme regulates intracellular processes linked to lipids accumulation and mobilization in normal tissues as well as in disease states, particularly cancer.

### 4.2. Functional Role of CK2 in de novo Fatty Acid Synthesis

**Figure 2:**
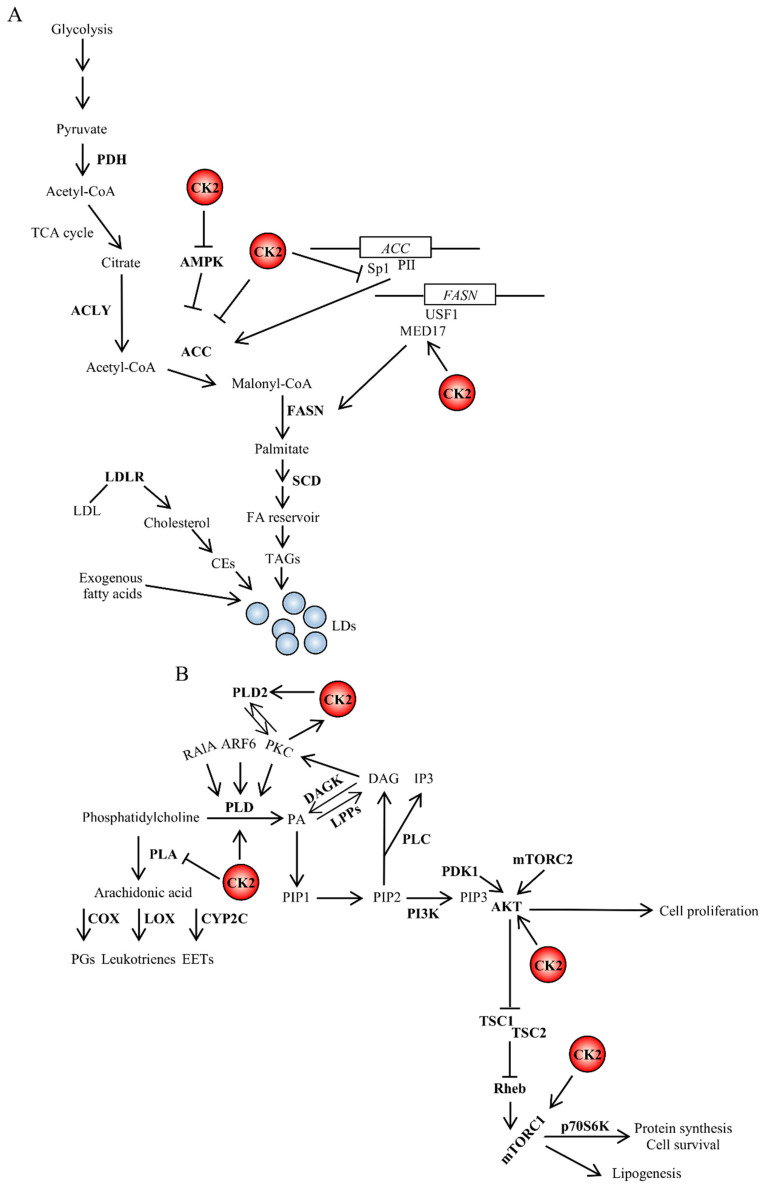
Crosstalk between CK2 and signaling pathways (**A**) regulating fatty acid synthesis or (**B**) controlled by phospholipases. CK2 is an important regulator of lipid homeostasis by stimulating fatty acid and protein synthesis and promoting cell survival. The interrelatedness between CK2 and lipid signaling occurs at multiple levels. The PI3K/AKT signaling pathway promotes lipid synthesis. Active AKT stimulates mTORC1, which, in turn, regulates several intracellular processes including cap-dependent translation, lysosomal biogenesis and lipid homeostasis. CK2 can directly phosphorylate AKT contributing to increase its catalytic activity [173]. Further details are discussed in the text. DAGK, diacylglycerol kinase; LPPs, lipid phosphate phosphatases; PA, phosphatidic acid; p70S6K, ribosomal protein S6 kinase beta-1; Rheb, Ras homolog enriched in brain; TSC1, tuberous sclerosis 1; TSC2, tuberous sclerosis 2.

De novo FA synthesis occurs in the cytoplasm and begins with the carboxylation of acetyl-CoA in the presence of NADPH and results in the production of malonyl-CoA. The enzyme ACC catalyzes this reaction that is considered the rate-limiting step in the synthesis of FAs. Two distinct ACCs exist in human tissues, i.e., ACC1 and ACC2 that are encoded by different genes. ACC1 is highly expressed in lipogenic tissues such as liver and adipose tissue, while ACC2 is mainly expressed in heart, muscle and to a lesser extent liver. Both enzymes are regulated at the transcriptional level as well as by post-translational modifications [152,153]. A phosphopeptide analysis of ACC was carried out in the attempt to identify the phosphorylation sites and the specific protein kinases able to control the activity of ACC. The study revealed eight different phosphorylation sites [154]. Among the identified enzymes, protein kinase CK2 was shown to phosphorylate ACC at Ser-29 following insulin stimulation in adipocytes; however, no changes in ACC carboxylase activity were reported (Figure 2A) [154,155,156]. The authors suggested that phosphorylation of ACC by CK2 could, in turn, promote the dephosphorylation of amino acid sites involved in the modulation of ACC enzyme activity. Indeed, this was plausible at that time as it was known that insulin treatment of fat cells increases the association of ACC to ACC-phosphatase [157]. Conversely, Zhang et al. provided evidence, partially supported by data from Armstrong et al. [158], that CK2 negatively regulates the expression of ACC. They reported that in pancreatic β-cells, high glucose levels increase the binding of specificity protein 1 (Sp1) transcription factor to the glucose response element of the PII promoter, thereby activating *ACC* gene expression. However, the glucose-mediated activation of PII expression is suppressed following the phosphorylation of Sp1 by CK2 resulting in decreased Sp1 DNA binding (Figure 2A) [159].

The functional role of CK2 in de novo fatty acids synthesis also includes the regulation of FASN a central enzyme in de novo lipogenesis that is mainly regulated at the transcriptional level by the organism’s nutritional status and in response to insulin [160]. Work carried out by Viscarra et al. showed that upstream transcription factor 1 (USF1), a key transcription factor for FASN activation, directly interacts with Mediator complex subunit 17 (MED17) at the FASN promoter. It was reported that MED17 is phosphorylated by CK2 in the liver of fed mice, and this event is required for lipogenesis. These findings, while adding more complexity to the transcriptional regulation of genes involved in USF1-mediated lipid synthesis, established an important link between FASN activation and CK2 which is required for the transcriptional activation of key lipogenic genes in response to insulin [161]. Analysis of the phosphorylation status of MED17 in leptin-deficient and insulin resistant, ob/ob mice also showed chronic phosphorylation of Ser-53, the CK2 target site. This suggested that the persistent phosphorylation of MED17 at Ser-53 might contribute to the chronic activation of lipogenesis and the onset of obesity in mice (Figure 2A) [161]. Although it is not clear whether the effect of CK2 on MED17 is specific to lipogenic genes, these findings raise the prospect that the target inhibition of this protein kinase could be a suitable option for the treatment of metabolic deregulation in health as well as disease states.

### 4.3. CK2 and the Intricate Networks Regulated by Phospholipases

The action of phospholipases on phospholipids leads to cleavage products that have an important role in intracellular signaling through the generation of precursors of signaling molecules. One of the earliest pieces of evidence of a crosstalk between CK2 and this class of enzymes derives from studies by Ganley et al. on the regulation of PLD1 activity [162]. The activation of PLD1 requires association with proteins and lipids. This includes ADP ribosylation factor (ARF) and Ras-related protein RAl-A (RalA), the conventional PKCα isoform and PIP_2_. PLD1 appears to contain several post-translational modifications in part catalyzed by PKC when present on the plasma membrane [163]. Ganley’s group showed that PLD1 is additionally phosphorylated by CK2 and interacts with this protein kinase. Changes in PLD1 cleavage activity were not reported; however, the existing evidence led to the speculation that CK2 might play an important role in signaling pathways mediated by PLD1 by stabilizing the phospholipase and facilitating its correct localization on cellular membranes [162]. Subsequently, experiments carried out employing human glioblastoma cells revealed that CK2 interacts with both PLD1 and PLD2, and that the CK2-mediated phosphorylation of PLD2 leads to its activation [164]. On the other end, the same research group was also able to demonstrate a reciprocal positive regulation between PLD2 and CK2, showing that PLD2 acts upstream of PKC, which directly stimulates CK2 activity through the phosphorylation of CK2α (Figure 2B) [165]. These results are in good agreement with earlier investigations from Yefi et al., who showed that CK2 is involved in the up-regulation of COX2 through the Wnt/β-catenin signaling pathway [166].

In contrast to these results, Han et al. showed that PLA1 stably associates with the different CK2 isoforms and the complex formation with CK2α results in 50% loss of catalytic activity of the phospholipase [167].

Overall, further studies are warranted to advance our knowledge on the crosstalk between CK2 and the family of phospholipase enzymes; however, the aforementioned findings suggest that CK2 might exert different effects that are isoform and/or tissue-specific and dependent on the biological context. In this respect, size exclusion experiments revealed the presence of large complexes of a soluble form of PLA1 containing CK2α in homogenates of the macaque testis, but not of the cerebral cortex, suggesting that the association between the aforementioned proteins may play a specific role in the differentiation of spermatids or in the function of sperm [167].

### 4.4. Crosstalk between CK2 and Insulin-Mediated Signaling

Insulin is a major endocrine hormone involved in the regulation of a plethora of biological processes including lipid metabolism and adipocytes development and differentiation, via the activation of intracellular signaling cascades downstream of the insulin receptor [168]. Insulin exerts a critical control of anabolic processes by facilitating glucose uptake, inhibiting intracellular lipolysis, promoting de novo fatty acid synthesis and triacylglycerides formation, enhancing the expression of various fat-specific transcription factors including SREBP1c and peroxisome proliferator-activated receptor gamma (PPARγ), and by modulating the activity of lipid-related enzymes such as lipoprotein lipase (LPL) [169]. Persistent stimulation of insulin release causes the body to produce excess fat, which may lead to obesity, posing the risk of developing several pathological conditions including type-2 Diabetes, cardiovascular diseases, respiratory abnormalities, and cancer [170,171]. The role of CK2 in glucose homeostasis is well documented and exerted at multiple levels, i.e., from the control of insulin expression and release to its role in pancreatic β-cell turnover. These and other aspects of the CK2-mediated control of endocrine pancreas physiology have been extensively discussed by Ampofo et al. in a recent review [172].

**Figure 3:**
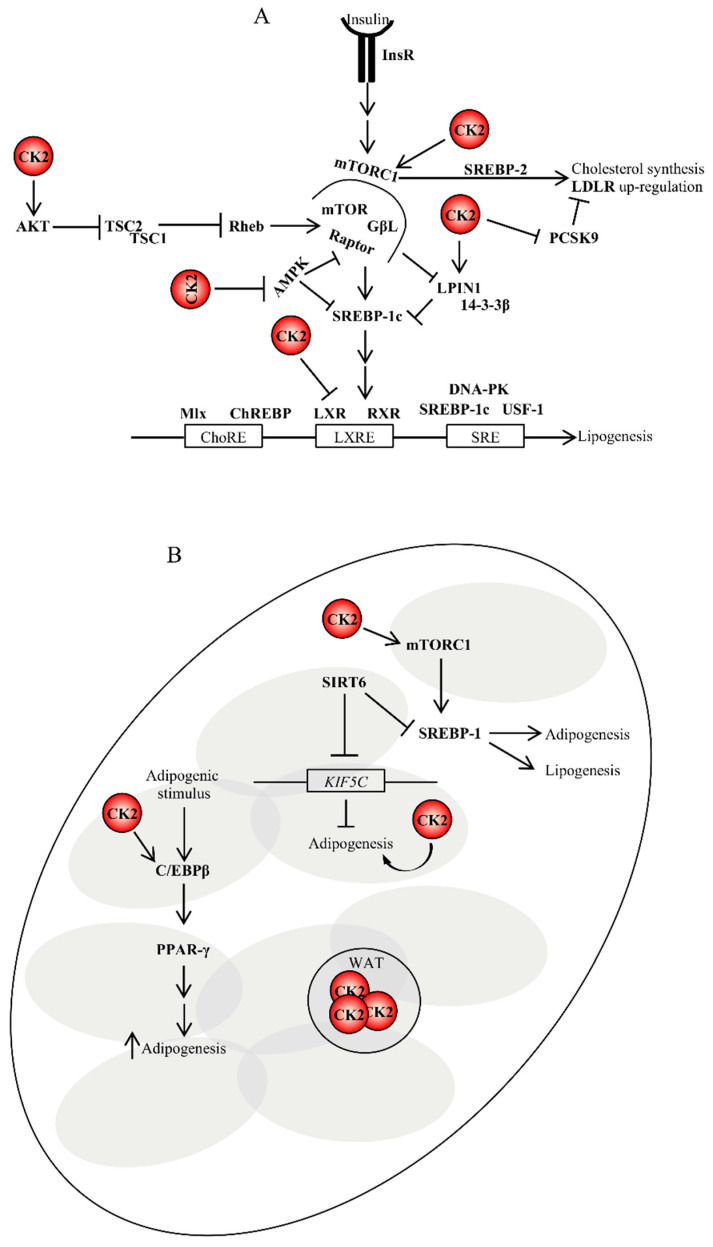
Role of CK2 in lipogenesis and adipogenesis. (**A**) CK2 controls lipogenesis at the translational levels. Insulin binding to its receptor on target cells results in the activation of lipid biosynthesis, which is a multi-step process regulated by key lipogenic enzymes. Their expression is controlled, at the transcriptional level, by the sterol regulatory element-binding proteins (SREBPs). To date, it is not known whether CK2 can directly regulate their activity; however, it does indirectly. (**B**) CK2 is also involved in the stimulation of adipogenesis in several ways as depicted in the figure and discussed in the text. For instance, CK2 positively regulates mTORC1 activity. mTORC1 upregulation stimulates the expression of SREBP1 promoting lipogenesis and adipogenesis. SIRT6 is an important regulator of lipogenesis as well. It represses SREBP1 by blocking its expression and processing [193]. SIRT6 is required for adipogenesis in vivo and in vitro. During adipogenesis, SIRT6 regulates mitotic clonal expansion of pre-adipocytes by repressing the expression of KIF5C and enhancing CK2 activity. ChoRE, carbohydrate response element; DNA-PK, DNA-dependent protein kinase; GβL, G protein beta subunit like; InsR, insulin receptor; LXRE, liver X-receptor response element; Mlx, Max-like protein; RXR, retinoid X receptor; SRE, sterol regulatory element; WAT, white adipose tissue.

The expression of genes involved in FA synthesis in the liver is regulated by insulin, which activates the transcription of SREBP1c through mTORC1 [174,175]. In addition, the insulin-mediated activation of SREBP1c transcription requires the complex formation between liver X receptor (LXR) and CCAAT/enhancer-binding protein beta (C/EBPβ) [176]. SREBP1c activity can be undermined by the nuclear accumulation of LPIN1 when the latter is not phosphorylated by mTOR [70]. A direct regulation of SREBP1 expression and/or activity by CK2 has not been reported; however, an indirect control is not be excluded. This is based on the fact that (i) CK2 phosphorylates LPIN1 at two distinct amino acids residues and lack of phosphorylation attenuates LPIN1 binding with 14-3-3β a regulatory protein that normally facilitates the cytoplasmic localization of LPIN1 [177], (ii) CK2 phosphorylates LXR and this post-translational modification modulates LXR transcriptional activity and restricts the range of LXR-responsive genes [178], and (iii) pharmacological inhibition of CK2 negatively affects the mTORC1 signaling cascade [179,180,181]. Additionally, AMPK represses the expression of SREBP1 target genes and the inhibition of CK2 up-regulates AMPK in vitro [181] and in vivo [182] (Figure 2B and Figure 3A).

Interestingly, mTORC1 has also been reported to up-regulate the expression of cholesterol biosynthetic genes by inducing SREBP-2 processing, which results in increased nuclear expression of the latter and subsequent LDL receptor (LDLR)-mediated cholesterol uptake [183]. Similarly, Ai et al. reported that mTORC1 increased LDLR protein expression by inhibiting pro-protein convertase subtilisin/kexin type 9 (PCSK9), a secreted protein which controls the degradation of LDLRs, resulting in decreased levels of circulating LDL cholesterol in vivo [184]. As in the case of SREBP-1, evidence that CK2 directly regulates the expression of SREBP-2 has not been provided. However, one could envisage that CK2 sustains the LDLR pathway by up-regulating the mTORC1 signaling cascade and/or blocking the maturation of PCSK9 by a posttranscriptional mechanism. This latter possibility is supported by studies by Dewpura et al., which demonstrated that a Golgi casein kinase-like kinase phophorylates pro-PCSK9 and suggested that this post-translational modification could protect the pro-peptide against proteolysis which is required for PCSK9 maturation [185].

Taken together, from the original observation that SREBPs are transcription factors playing different roles in lipid synthesis, it is now accepted that these signaling molecules are master regulators of lipogenesis in various physiological and pathological processes. Their regulation occurs at multiple levels and CK2 plays a part in it.

### 4.5. CK2 Is Implicated in the Regulation of Pre-adipocytes Differentiation into Adipocytes

Wilhelm et al. showed that pharmacological inhibition of CK2 suppresses the differentiation of murine 3T3-L1 cells into adipocytes [186]. In an effort to elucidate the underlying molecular mechanisms, several independent research groups demonstrated that (i) CK2 positively regulates the activity of C/EBPβ an upstream transcription factor of PPAR-γ2 whose nuclear localization is controlled by CK2 in the early phases of pre-adipocytes differentiation [187,188,189], (ii) SIRT6 deacetylase, which is an essential factor for mitotic clonal expansion during adipogenesis, represses the expression of kinesin heavy chain isoform (KIF5C), a negative regulator of adipogenesis, and (iii) reduction in KIF5C, which is a binding partner of CK2, results in CK2 nuclear translocation and, thus, the induction of mitotic clonal expansion (Figure 3B) [190,191,192]. The crosstalk between CK2, sirtuins (SIRTs) and metabolism will be further discussed in this review.

Studies conducted on rodents and humans demonstrated the presence of at least two distinct populations of thermogenic adipocytes, which respond to certain external stimuli, i.e., brown adipocytes and beige adipocytes—the latter are scattered within white adipose tissue [194]. As further discussed in this review, the phosphoproteomic analysis of brown, beige and white adipocytes stimulated with norepinephrine revealed the activation of CK2 in white adipocytes, while its inhibition with small-molecule compounds promoted beige adipocytes biogenesis and protection from diet-induced obesity and insulin resistance in mice [195].

Overall, these data provide important insight into the physiological role of CK2 with respect to obesity and the adipocytes-mediated control of thermogenesis, and support the notion that the pharmacological inhibition of this enzyme represents an attractive therapeutic option to combat obesity and disorders linked to it.

### 4.6. Adipocytes, Adipocytokines, Cancer Risk and CK2

Several epidemiological studies support a link between specific circulating adipocytokines and cancer risk. This is in part due to the fact that adipose tissue undergoing excessive lipid accumulation releases hormones, growth factors and pro-inflammatory cytokines, resulting in chronic systemic low grade inflammation [196]. After the discovery of leptin [197], many other adipokines have been identified including adiponectin, resistin, tumor necrosis factor-α (TNF-α), interleukin 6 (IL-6) and apelin, creating a challenging complexity in the field [198,199]. Evidence of a crosstalk between CK2 and cytokines has been provided by Harris et al., working on interferon-γ (IFN-γ)—a key regulator of the immune and inflammatory response in different tissues including the adipose tissue. They showed that the IFN-γ-mediated inhibition of LPL promoter-activity was prevented by the expression of dominant negative forms of CK2 and AKT [200]. CK2 has also been linked to the leptin-mediated regulation of PTEN. Ning et al. demonstrated that this cytokine increases the phosphorylation of PTEN at multiple residues in a CK2- and GSK3-dependent manner [201]. A crosstalk between CK2 and adiponectin-mediated signaling has been shown in studies aiming at elucidating signaling pathways downstream of the adiponectin receptors 1 and -2 (i.e., AdipoR1 and -R2). A yeast two-hybrid-based approach supported by other biochemical methodologies led to the identification of CK2 as a binding partner for AdipoR1 and an effector molecule in adiponectin-dependent pathways at the crossroad between adiponectin and insulin signaling cascades [202,203].

Adipocytokines comprise true mediators produced in the adipose tissue as well as cytokines also produced by other cell types. IL-6 is an example of cytokines secreted by different tissues. It possesses strong pro-carcinogenic activity and its levels were found to negatively correlate with the prognosis of patients affected by cancer including breast, lung and colon [204,205,206,207]. Hence, it is not surprising that much effort has been devoted to identifying strategies to disrupt its activity and/or synthesis. A possible link between CK2 and IL-6 has been investigated in connection with inflammatory breast cancer (IBC). Work carried out with IBC cell lines demonstrated that pharmacological inhibition of CK2 blocks IL-6 secretion. Most importantly, the outcome of a Phase I clinical trial indicated that a patient with inflammatory breast cancer treated with CX-4945, a potent and selective inhibitor of CK2 [151], displayed substantially reduced levels of plasma IL-6 [208]. Given the limited in vivo evidence, the mode by which CK2 regulates IL-6 secretion remains to be seen; however, these data position CK2 inhibition as an effective strategy to block deregulated secretion of IL-6 in pathological situations.

Taken together, the potential molecular mechanisms underlying the reported observations have not been comprehensively elucidated in many instances, nevertheless, the findings altogether support the notion that CK2 plays a central role in mediating critical cellular responses linked to adipogenesis and adipose tissue biology in health and disease states.

### 4.7. Crosstalk between CK2 and SIRTs in Obesity and Cancer

According to the World Health Organization, overweight and obesity are defined as abnormal or excessive fat accumulation that present a risk to health. Lifestyle and genetic factors are some of the main reasons for the development of obesity [209,210]. Sirtuins (SIRTs), which are composed of seven members (SIRT1-SIRT7), are histone/protein deacetylases [211]. SIRT1 is a NAD+-dependent deacetylase that functions as a master energy sensor and is involved in many obesity-related diseases including non-alcoholic fat liver disease (NAFLD) [212]. Under a systematic investigation for post-translational modifications of SIRT1, Zschoernig et al. identified two CK2 sites targeting Ser-659 and Ser-661 in human cells in vitro and in vivo [213]. The authors speculated that the phosphorylation might be of physiological relevance. In the same year, Kang et al. showed that ionizing radiation in murine cells leads to CK2/SIRT1 interaction and SIRT1 activation [214].

Choi et al. demonstrated that Ser-164 is a major serine phosphorylation site in obese, but not lean, mice [212]. This phosphorylation was catalyzed by the protein kinase CK2, the expression of which is elevated in obesity. Later on, it was shown that the phosphorylation of SIRT1 at Ser-164 inhibited its nuclear localization and affected in part its deacetylase activity [215]. Ser-164 is part of a typical CK2 recognition site, i.e., S164SSD. The canonical CK2 recognition peptide site is SXXD/E. It is of interest to note that the phosphorylation sites responsible for SIRT1 activity are located at the C-terminal end of the protein [214], whereas the phosphorylation which is associated with obesity is located at the amino terminal end [212].

Obesity as part of the metabolic syndrome is a rapidly growing epidemic which increases the risk for the development of chronic inflammation, diabetes, hypertension, coronary heart disease, hyperlipidemia, cancer and other. Thus, the inhibition of SIRT1 phosphorylation catalyzed by CK2 may serve as a new therapeutic approach for treatment of obesity-related diseases.

In another investigation, in mouse models of obesity, Borgo et al. [215] showed that both the amount and activity of CK2 are substantially higher in white adipose tissue (WAT) of ob/ob and db/db mice than of controls, while they are similar in brown adipose tissue (BAT), muscle and liver. These results are consistent with a report showing that CK2 is preferentially activated in high-fat diet mice [195]. Chronic inhibition of CK2 by small-molecule compounds for forty days has been shown to protect mice from diet-induced obesity. Interestingly, CK2 protein and activity levels are greatly up-regulated not only in WAT from ob/ob and db/db mice, but also in obese patients. Weight loss obtained by hypocaloric diet reverted CK2 hyper-activation to a normal level. Therefore, inhibition of CK2 may open a new therapeutic approach to target human obesity [215].

How to tackle obesity? A straightforward strategy to treat obesity is via increased physical activity and food restriction [216]. Especially, the consumption of foods rich in bioactive anti-inflammatory compounds such as omega-3 fatty acids and polyphenols has been documented to reduce inflammation [217,218]. Cellular studies demonstrated that dietary polyphenols such as resveratrol, curcumin, etc., exerted beneficial effects on lipid and energy metabolism and potential body weight change [219,220]. In particular, several studies in vitro and in animal models have shown promising anti-inflammatory and antioxidant effects of resveratrol in liver cells. These effects seem to be mainly mediated by the activation of the signaling pathways of SIRT1 and AMPK and the inhibition of the NFκB pathway [221]. Significant effects of resveratrol were observed with dietary interventions and promotion of physical exercise, and by improving the absorption of resveratrol through micronized application [222,223,224]. These results provide significant support for the idea that resveratrol might protect against the metabolic syndrome and type-2 Diabetes by activating SIRT1 [219,220,221,225,226].

In another report on SIRT/protein kinase CK2, Bae et al. [227] reported the phosphorylation of Ser-338 in SIRT6, catalyzed by protein kinase CK2. Overexpression of SIRT6 in breast cancer cells increased proliferation, but mutation at the CK2 Ser-338 phosphorylation site of SIRT6 inhibited the proliferation of breast cancer cells. This does not come as a surprise, since protein kinase CK2 has been shown to be overexpressed concomitantly with an increase in activity in many different human cancers [134]. Taking everything into consideration, their study shows that CK2 and SIRT6 are indicators of poor prognosis for breast carcinomas and that CK2-catalyzed phosphorylation of SIRT6 might be involved in the progression of breast carcinoma [227].

## 5. Conclusions

Today, the Warburg effect matters more than ever. Some of Otto Warburg’s assumptions turned out to be not true, but he was right in emphasizing the importance of metabolic alterations in cancer cells. The metabolic reprogramming seen in cancer cells derives from altered oncogenes and tumor suppressor genes, and it is a process essential to fuel and maintain the uncontrolled growth of cancer.

Besides understanding the role of altered glycolytic pathway in cancer cells, researchers are focusing more and more on other critical metabolic processes including nucleotide and lipid biosynthesis. The concept of targeting cell metabolism is not new, but the idea is to look at this phenomenon with different lenses. The field of lipid metabolism and its aberration in cancer is complicated, and much work remains to be done regarding our understanding of the crosstalk between lipid metabolism pathways and the tumor microenvironment, which is mostly hypoxic. Different cancer cell types show various degrees of metabolic reprogramming for thriving in adverse conditions imposed by hypoxia and the deprivation of exogenous lipids and other nutrients. This metabolic adaptation, now considered a hallmark of cancer, renders cancer cells highly dependent on de novo lipid synthesis. Hence, pharmacological targeting of key enzymes contributing to the synthesis of these essential macromolecules could have important implications in limiting the proliferation of cancer cells.

Protein kinase CK2 is a pleiotropic enzyme linked to a variety of cellular functions. The strength of this enzyme lies in its ability to regulate multiple signaling cascades as a holoenzyme or through its individual isoforms. CK2 is undoubtedly implicated in the control of lipid metabolism at multiple levels in health as well as disease states. It has been reported that CK2 contributes to increasing adipocyte hyperplasia/hypertrophy and it is overexpressed in obese patients. Hence, pharmacological inhibition of this enzyme might be an attractive approach to combat obesity and improve the care of obesity-associated diseases.

The up-regulation of CK2 has been invariably detected also in cancer. Hence, it should come as no surprise that this enzyme also helps to sustain elevated rates of growth in malignant cells by controlling enzymes regulating key steps in the signaling pathways involved in lipogenesis.

The role of CK2 with respect to the regulation of lipid metabolism is far from complete. With the advent of more sophisticated technologies, we will certainly understand more and more about the complexity of metabolic reprogramming in cancer and the role of CK2 in regulating this process. The study of lipid metabolism is now at the forefront of cancer research and CK2 is a part of it. In light of its implication in lipid metabolism, future therapeutic strategies should take into account novel dietary interventions combined with the pharmacological targeting of CK2 as a novel antineoplastic approach.
